# Supplementary material for: The causal relationship between the human gut microbiota and pyogenic arthritis: a Mendelian randomization study
Source: Front Cell Infect Microbiol. 2024 Nov 26;14:1452480. doi: 10.3389/fcimb.2024.1452480 (PMC11629706; doi:10.3389/fcimb.2024.1452480)
Supplement: Supplementary file 1 [file DataSheet1.zip › mendelian test/ebi-a-GCST90027618.csv_forest.pdf]

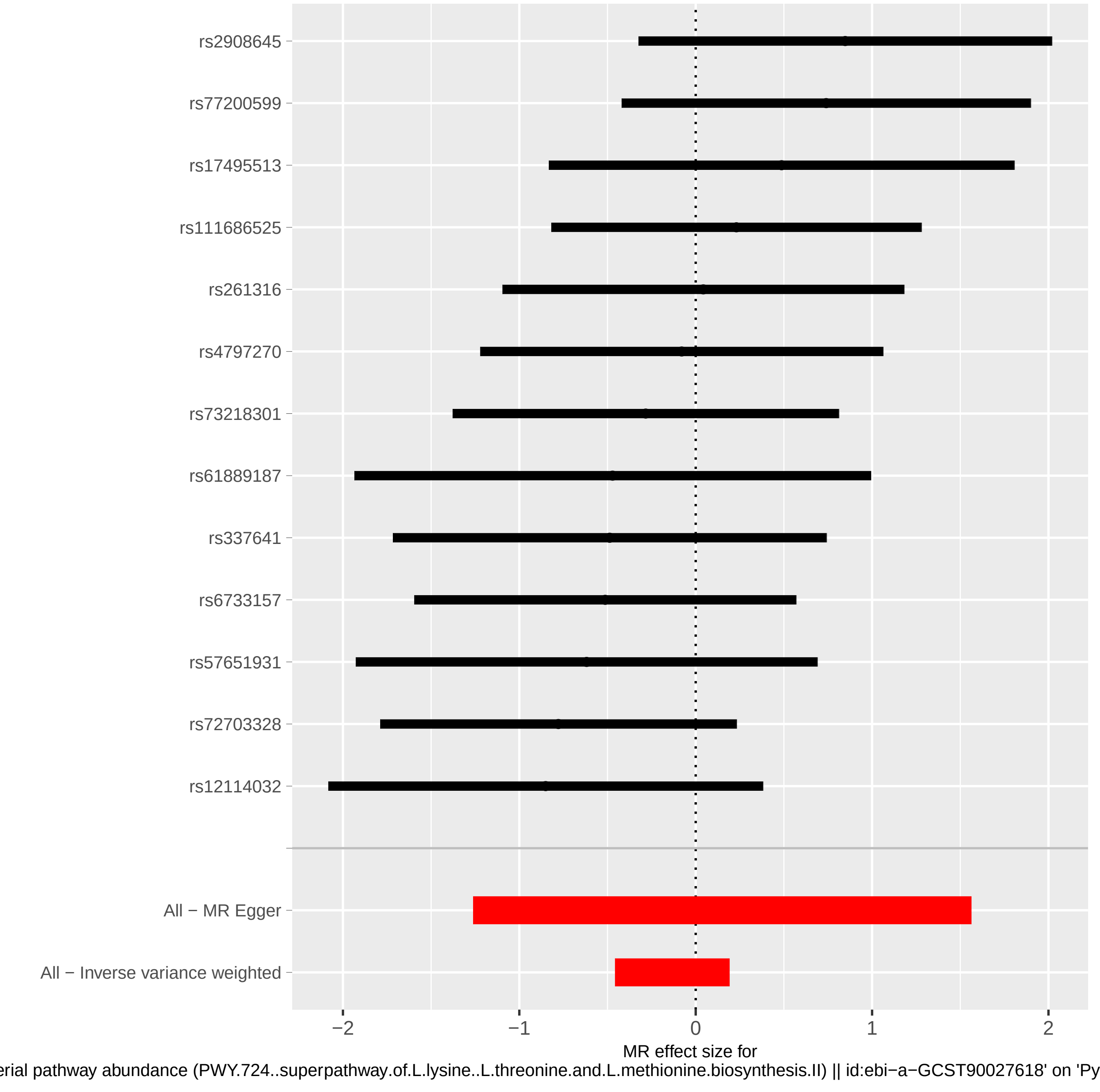
rs2908645
rs77200599
rs17495513
rs111686525
rs261316
rs4797270
rs73218301
rs61889187
rs337641
rs6733157
rs57651931
rs72703328
rs12114032
All − MR Egger
All − Inverse variance weighted
−2
−1
0
1
2
MR effect size for
erial pathway abundance (PWY.724..superpathway.of.L.lysine..L.threonine.and.L.methionine.biosynthesis.II) || id:ebi−a−GCST90027618' on 'Py
